# Planning influenza vaccination programs: a cost benefit model

**DOI:** 10.1186/1478-7547-10-10

**Published:** 2012-07-26

**Authors:** Ian G Duncan, Michael S Taitel, Junjie Zhang, Heather S Kirkham

**Affiliations:** 1 Clinical Outcomes & Analytic Services, Walgreens Co., 1415 Lake Cook Rd., MS L444, Deerfield, IL, 60015, USA

**Keywords:** Influenza, Immunization, Vaccination, Cost benefit, Economic model

## Abstract

**Background:**

Although annual influenza vaccination could decrease the significant economic and humanistic burden of influenza in the United States, immunization rates are below recommended levels, and concerns remain whether immunization programs can be cost beneficial. The research objective was to compare cost benefit of various immunization strategies from employer, employee, and societal perspectives.

**Methods:**

An actuarial model was developed based on the published literature to estimate the costs and benefits of influenza immunization programs. Useful features of the model included customization by population age and risk-level, potential pandemic risk, and projection year. Various immunization strategies were modelled for an average U.S. population of 15,000 persons vaccinated in pharmacies or doctor’s office during the 2011/12 season. The primary outcome measure reported net cost savings per vaccinated (PV) from the perspective of various stakeholders.

**Results:**

Given a typical U.S. population, an influenza immunization program will be cost beneficial for employers when more than 37% of individuals receive vaccine in non-traditional settings such as pharmacies. The baseline scenario, where 50% of persons would be vaccinated in non-traditional settings, estimated net savings of $6 PV. Programs that limited to pharmacy setting ($31 PV) or targeted persons with high-risk comorbidities ($83 PV) or seniors ($107 PV) were found to increase cost benefit. Sensitivity analysis confirmed the scenario-based findings.

**Conclusions:**

Both universal and targeted vaccination programs can be cost beneficial. Proper planning with cost models can help employers and policy makers develop strategies to improve the impact of immunization programs.

## Background

Seasonal influenza (flu) is a costly disease to patients, employers, and society in terms of direct and indirect medical cost and lost productivity. The most recent estimates of the cost of flu from 2003 showed $10.4 billion annually in direct medical costs and $16.3 billion in indirect costs associated with lost earnings and loss of life [1]. From a societal perspective, the total economic burden of the flu in the United States is $87.1 billion [1]. During influenza season, influenza-like-illness (ILI) is responsible for 45% of workdays lost and for 49% of low productivity days among working adults aged 50–64 years [2].

Although vaccination is effective in decreasing the burden of influenza, immunization rates are below recommended levels [3]. As of the 2010 influenza season, the Centers for Disease Control and Prevention (CDC) recommended that everyone 6 months of age and older should be vaccinated against the flu as soon as a seasonal vaccine is available [4]. Recent evidence from the Canadian health system asserts that universal vaccination decreased the burden of ILI [5,6]; however, concerns remain in the United States about whether such efforts will be cost effective and whether there are enough immunization providers to achieve universal vaccination [7]. Immunizations were traditionally delivered via three channels: (a) physicians in their offices, (b) public health systems in a number of settings such as community health clinics and schools, and (c) in hospitals [8]. In the mid-1990’s, national programs were developed to train pharmacists to provide immunization services [9]. The H1N1 pandemic in 2009 and the call for universal vaccination in 2010 further highlighted the need for immunization providers and the value of pharmacy-based vaccinations.

Vaccination coverage is defined as the percentage of persons in a population who have received at least one dose of influenza-containing vaccine in a given year. To improve vaccine coverage in the United States, a Report of the National Vaccine Advisory Committee [10] advocated vaccinations in nontraditional settings such as pharmacies and workplaces. Compared to traditional channels such as physician offices, nontraditional settings can improve access, increase convenience, and decrease cost [8]. Furthermore, the CDC recognizes the opportunity of collaborating with employers and businesses to promote vaccination [11]. Employers can reduce both access and cost barriers to vaccination by offering on-site immunization, designing a benefit plan that minimizes out-of-pocket cost, or providing financial incentives for community-based immunization.

When businesses wish to evaluate the allocation of resources, they often build models to aid in decision-making. Several researchers have published economic models to evaluate either the cost or benefits of seasonal influenza vaccination, or both [2,12,13]. No published model was found which (a) allowed decision makers to project outcomes for a specific target population, (b) aggregated different models to account for a wide array of immunization strategies, (c) allocated savings to multiple stakeholders, or (d) trended value to the current year. Therefore, we developed a model that combines components from the published literature in a flexible yet comprehensive approach. The actuarial model allows users to estimate the costs and benefits of any community or worksite immunization program. The resulting value of immunization was evaluated from three perspectives: an employee, an employer, and society. When vaccination prevents influenza, employees may have reduced expenses, improved quality of life, and possibly reduced wage-loss. If their employees remain healthy, employers could benefit from less absenteeism, higher productivity, and lower healthcare expenses, which could offset the cost of the immunizations. Society benefits from reduced influenza cases by avoiding disruption to business and communities that occurs from either seasonal or pandemic infections. While this model uses the terminology of employee and employer, employer savings could be applied to other payers such as health plans or government payers such as Medicare due to the flexibility of variables. Likewise, savings apportioned to employees could equally accrue to members of a health plan. Because the cost of delivering vaccinations (and associated cost sharing) differs by channel, the savings model was developed to allow users to compare different delivery channels. Therefore, the objective of this research was to compare cost benefit of various immunization strategies from the perspective of these three stakeholders.

## Methods

### Model overview

The cost benefit model was designed to evaluate the costs, benefits, and net savings associated with influenza immunizations. It is used to illustrate the financial value of a program to a set of stakeholders over a specific time period. Sensitivity testing is performed to vary the user input and model assumptions to make sure the best model assumptions are used to generate the most reasonable the financial value projections. We used an actuarial approach because it allows for multiple interrelated assumptions around various aspects of risks for a set of contingent future events to determine the most reasonable time value of money for different stakeholders [14]. The model provides users with a base set of assumptions and variable values derived from literature but with considerable ability to vary those assumptions interactively for specific populations. Key variables that affect outcomes are summarized in Table1 and organized into inputs, cost categories, and outputs. The majority of cost assumptions were taken from two studies: Molinari et al. [1] and Prosser et al. [12]. Both studies used Monte Carlo simulations based on the same retrospective analysis of influenza-related utilization over multiple years from a large database of insurance claims (Medstat Marketscan) to model costs of influenza. 

**Table 1:** Summary of model variables

**Inputs**	**Cost Categories**	**Outputs**
· Projection year	· Hospitalization	· Number of members immunized
· Client population demographics (i.e., age and dependent profile)	· Outpatient visits	· Expected cost of immunizations
· Vaccination delivery channel	· Self-medication	· Expected saving total
· Percentage vaccinated	· Absenteeism	· Stakeholder allocation
· Economic variables (e.g., average value of workday lost)	· Presenteeism	· Expected net saving total
· Benefit design (e.g., flu vaccine cost-sharing policy)	· Death	· Expected net savings per vaccinated (PV) member
· Vaccine effectiveness		· Expected net saving per member per year (PMPY)
· Risk Level		
· Risk of pandemic		

Molinari et al. first used national epidemiological data and published studies to estimate the probability of four categories of influenza health outcomes: illness, outpatient visits, hospitalizations, and death. All probabilities were age and risk stratified. Next, Molinari and colleagues estimated direct and indirect costs associated with influenza. Most direct costs were based on 179,718 cases of influenza identified in the Medstat Marketscan database between 2000 and 2004. Indirect costs valued lost productivity due to illness or death. Monte Carlo simulations with sensitivity analysis yielded estimated cases and average costs (with 95% upper and lower confidence intervals). The probabilistic model predicted an estimated 30,151,934 annual cases that resulted in 21,354 [22,636, 43,507] outpatient visits, 3,131 [2,108, 4,511] inpatient days, 44,003 [25,694, 62,484] lost productivity days, and 611 [360, 953] undiscounted life years lost. Total direct costs and total economic burden in 2003 dollars was estimated as $10.4 billion [$4.1, $22.2] and $87.1 billion [$47.2, $149.5], respectively.

Molinari et al. did not project potential savings for averted cases due to vaccination; however, Prosser et al. built on Molinari’s cost assumptions to estimate cost savings of vaccination in various clinical settings, such as mass vaccination events, pharmacy and doctors’ offices. Prosser et al. noted that non-traditional settings, such as pharmacies, were highly cost effective. While their results provided general guidance for comparative cost efficacy, estimating cost efficacy for a particular population would require some effort. Hence, we built upon the foundation of both previous studies to build a decision support tool to model various immunization strategies.

Inputs

1. **Projection Year:** Because of medical inflation, cost variables will differ by calendar year. The underlying assumptions and factors in the model are derived from different years and inflation factors are used to adjust the literature-based variables to project to the period chosen by the model user. Medical costs were adjusted by medical trend, while non-medical costs were adjusted by the consumer price index. The default projection year is the next (i.e., upcoming) influenza season.

2. **Population demographics:** Risk of influenza and its complications varies by age. While some populations (e.g., employers) will be more heavily weighted towards younger persons as employers terminate their retiree benefits, other populations such as a Medicare health plans may be older. Therefore, the model allows users to tailor savings estimates to the specific demographics of the client population. Five age groupings are used in the model: ≤11 (children), 12–17 (youth), 18–49 (young adults), 50–64 (middle-age adults), and 65 and older (older adults). A sample population of 15,000 persons is provided as a default in the model with a typical age profile (13%, 13%, 40%, 20%, and 13%, by respective age group), based on a 2009 estimate of the U.S. census [15].

3. **Vaccination delivery channel:** By surveying non-traditional immunization providers, Prosser et al. [12] found that the immunization costs were lower at mass vaccination events and pharmacies compared to traditional channel (TC) settings. To compare costs across channels, three input variables were needed: cost in traditional settings ($_TC_), cost in non-traditional settings ($_NTC_), and the proportion of persons using non-traditional channels (%_NTC_). To provide a default value for traditional channels, we derived an average allowed charge per physician administered influenza vaccination ($_TC_ = $82) from a large national dataset [Unpublished data from Solucia Consulting's proprietary database]. The model also allows users to adjust the expected utilization of non-traditional settings (default %_NTC_ = 0.50) and the cost of immunizations in such settings (default $_NTC_ = $30).

4. **Percent vaccinated:** As evidenced in the literature, not everyone who should get a flu shot actually gets one [16]. Coverage rates not only remain below national targets but also vary substantially by age group. To account for this variation, the model permits users to estimate the percent of population who are successfully vaccinated by age group. The model provides default coverage rates by age group (< 11: 55.2%, 12–17: 55.2%, 18–49: 49%, 50–64: 49%, 65+: 72%) based on U.S. coverage during the 2009/2010 season [16]. Alternatively, users can enter their own projected coverage distribution. Of note, this measure of adherence is distinct from vaccine effectiveness, which is discussed later in this section.

5. **Economic assumptions**: Economic assumptions include productivity and wages. The model provides baseline numbers based on U.S. averages [15], but allows the user to vary these assumptions to meet the needs of a specific population. To increase precision of productivity and death cost estimates, three inputs are required to calculate the average value of a lost workday: (a) Current Average Annual Wage, (b) Ratio of Benefits to Wages, and (c) Assumed Workdays per Year. The default assumption is an average annual salary of $40,000 with a 250 workday year increased by 30% to accommodate the value of employee benefits.

6. **Benefit design:** By providing details of cost sharing of vaccination and other medical services, the model is able to allocate costs and benefits to employers and employees. The default values assume that the plan (as funded by the employer) covers 90% of the vaccination costs and 78% of general medical costs [17]. When the benefit design for a particular population is known and different from the default value, the model can more accurately portray the distribution of cost and savings estimates to stakeholders.

7. **Preventable cases:** The proportion of ILI cases that can be prevented by vaccination is contingent on the *effectiveness of vaccine* and the *incidence of disease*. Effectiveness of the seasonal influenza vaccine varies by year, depending on many factors including the infectivity, pathogenicity, and virulence of the viral strains and the antigenic match with the vaccine. Furthermore, effectiveness varies by age group (due to human behaviors and social networks as much as characteristics of the virus) or co-morbid risk factors (due to decreased immunity). Interconnected with effectiveness, incidence also varies by strain and by age group. Two published studies [12,18] combined these concepts of vaccine effectiveness and influenza incidence into a rate of avoidable cases that was used in our model. The proportion of avoidable cases was higher in children aged 5–11 (5.5%) and persons aged 65 and older (5.4%) compared to youth aged 12–17 years (4.1%), young adults aged 18–49 years (4.6%), or middle-aged adults 50–64 (4.6%). For our model, estimated avoided cases among children under 5 years were assumed to be similar to children aged 5–11. These figures were based on non-pandemic years. To account for greater infectivity and severity during pandemic years, the model incorporates a pandemic risk factor, which is discussed in a later section.

8. **Risk Level:** Individuals at higher risk for ILI and its complications are more likely to incur ILI-related expenses compared with individuals with lower risk. Several published studies stratified cost assumptions by risk category. Therefore, the model allows one of two immunization strategies based on risk level: (a) immunizing the entire population (i.e., universal vaccination) or (b) targeting at-risk individuals. The definition of *high risk* followed Molinari et al. [1], who defined high risk as the presence of at least one co-morbid condition, as listed in Advisory Committee on Immunization Practices (ACIP) guidelines [4]. The risk profile by Molinari et al. estimated the proportion of high-risk individuals among age 17 and under, aged 18–49 years, 50–64 years, and 65 and older as 10.6%, 14.9%, 33.0%, and 51.2%, respectively. Although the Molinari profile was published between 1994 and 1997, this distribution was used because it remains consistent with 2010 CDC reported risk strata: 8.6% of youth (aged 12–17 years), 15.2% of young adults (aged 18–49 years), and 33.5% of middle-aged adults (aged 50–64 years). To be consistent with current ACIP guidelines, our model assumed all persons aged 65 and older were considered at high risk of influenza infection and complications [4]. The difference between a population and high-risk strategy is the type of individuals *targeted* for vaccination. Such strategies do not assume that all targeted individuals *received* the vaccination; therefore, coverage rates were also applied to high-risk vaccination programs.

9. **Risk of Pandemic:** During years with pandemic influenza, the value of vaccination is greater because the risk of exposure and infection is higher compared to non-pandemic years [13]. Based on the available data from the last three pandemics, Crowe and colleagues [19] estimated that there is a 2% probability of a pandemic (PrP = .02) in any given year, and pandemic flu incidence ranged from 9% to 35% , compared to 4% to 6% in non-pandemic years. Since incidence of pandemic influenza continues to vary with age, an attack rate adjustment (ARA) was used to tailor risk for the age distribution in the model:

(1) ARA=pandemic incidence/age−weighted average nonpandemic incidence

To account for the potential of a pandemic in any given year, utilization costs were adjusted by a pandemic risk adjustment factor (PRA), whereby:

(2) $$\text{PRA}=\left(1–\text{PrP}\right)*1+\text{PrP}*\text{ARA}$$

### Cost categories

Influenza and its complications impose costs on patients, health plans, employers, and society. The literature identified six categories of potential costs associated with a diagnosis of influenza: inpatient hospitalization, outpatient visits, self-medication, death, and productivity (presenteeism and absenteeism).

1. **Hospitalization:** Molinari et al. [1] defined costs for the hospitalization episode to include inpatient costs as well as outpatient and pharmacy services incurred 2 weeks prior and 4 weeks after the inpatient stay. Using Molinari’s definition, Prosser et al. reported hospitalization costs by age and risk. For example, among persons without risk factors for influenza, hospitalization was most costly for persons who were aged 50–64 years (Mean = $23,281; Range: $20,887-$26,159) [12]. This model used Prosser’s 2004 mean estimates, which were trend adjusted for the projected program year.

2. **Outpatient visits:** As one of the most frequent costs of influenza, outpatient services were defined by Molinari et al. as visits to primary care providers, specialists, urgent care clinics, and emergency rooms for an ILI. In addition to the cost of the visit, any pharmacy and laboratory services within a 3-day window were also included to estimate total outpatient costs not associated with inpatient episodes. Like hospitalizations, the range of outpatient costs were age and risk dependent (range = $51-$765 per person with influenza) [12,18].

3. **Self-medication:** Use of over-the-counter (OTC) medications is an early indicator of disease because many patients will use OTC either in lieu of or in addition to seeking medical care [20]. The cost of the typical "flu basket" was used to provide an approximation of the costs of self-medication. The flu basket includes products that are sold along with purchase of an OTC flu medicine, such as cough drops, pain relievers, decongestants, or juice. A retrospective analysis of data from a large pharmacy chain was used to estimate the average value of the basket in 2010.

4. **Death:** In the model, the costs associated with death were based on the value of a statistical life (VSL), which is a commonly used and comprehensive estimate to represent the “lost productivity as well as the intrinsic, or social, value place on human life” [1]. To calculate the savings from death avoided due to influenza vaccination, costs associated with death (as reported by Spurr in Molinari et al.) [1] were multiplied by the proportion of preventable deaths (as reported by Grosse in Prosser et al.) [12,18].

5. **Absenteeism and presenteeism:** In the United States, presenteeism and absenteeism due to influenza accounts for approximately 17 million lost workdays per year [21]. 

a. *Absenteeism* is defined as days taken off work to care for influenza illness in oneself, a child, or other dependent. In the Molinari study [1], estimated lost workdays ranged from 0.5-1.0 days in non-treated ILI, 1–7 days in outpatient-treated ILI, or 8–24 days in ILI requiring hospitalization. Absenteeism due to child’s illness ranged from 0.7-2.8 days per episode of flu. While this latter estimate seemed low, the estimate may be reasonable given that only one parent in a two-parent household would take time off.

b. *Presenteeism* was defined as decreased productivity due to workers with influenza continuing to be present in the workplace [22]. Presenteeism was estimated from the average reduced work effectiveness days as reported by Nichol [23].

Estimated days lost due to either absenteeism or presenteeism was transformed into costs by multiplying by the calculated average daily wage of the population, as discussed in the previous input section.

### Model outputs

Our basic cost model estimates net savings as total costs avoided due to disease prevention from vaccination minus the total costs of vaccination. This calculation is comparable to that reported by Nichol et al. [23] but provides cost output as a positive savings rather than negative costs.

1. **Number of persons immunized (NPI)** was calculated from the input number of individuals in the population in an age category, multiplied by the expected coverage in each age category, and totaled across all age categories. As previously discussed, the default vaccination rates are based on national coverage estimates by age. When a high-risk strategy is selected (HR = 1), NPI also accounts for the proportion of individuals who were estimated to be high risk by age group. Therefore:

(3) $$\text{NPI}=\Sigma \left(n_{\text{i}}*{\text{V}}_{\text{i}}\right)*\left(\text{if HR}=1,{\text{HR}}_{\text{i}}\right),1)\text{,}$$

where *n* = subset of persons in the i^th^ age category, with the corresponding estimates of coverage (V_i_) and high risk (HR_i_).

2. **Expected total immunization cost (COSTS)** for a non-traditional channel (NTC) such as pharmacies and a traditional channel (TC) such as doctor’s offices were calculated as a product of the unit cost of vaccination ($) in that channel multiplied by the count of number of persons vaccinated in each channel. Therefore, total costs were estimated as:

(4) $$\left({\$}_{\text{NTC}}*n_{\text{NTC}}\right)+\left({\$}_{\text{TC}}*\left(1-n_{\text{NTC}}\right)\right)\text{,}$$

where *n*_NTC_ = NPI * percent of immunizations in non-traditional channels.

These assumptions were discussed in the input section (3).

3. **Total expected savings (SAVINGS)** from avoided influenza cases are derived, for each risk and age category, from the product of (a) the number of persons immunized, (b) the estimated preventable cases per person immunized, (c) the unit of utilization per preventable case for each cost category, and (d) the unit cost for each cost category. For example, the equation for savings from avoided inpatient (IP) utilization is:

(5) $$SAVING(IP)=NPI*\frac{\left(Preventable\;flu\;cases\right)}{\left(Person\;immunized\right)}*\frac{IP}{Flu\;case}*Average\;flu\;IP\;cost$$

Parallel equations were constructed for outpatient, medication, and productivity savings. Total savings was the sum of these potential savings, in addition to estimated savings from averted deaths, which is discussed in the next section.

4. **Stakeholder allocation:** In the model, vaccination cost and savings are allocated to three stakeholders: payers (e.g., employers, health plans, or government payers), individuals (e.g., health plan members or employees and their dependents), and society (e.g., communities, families).

a. Total *costs of vaccination* were distributed to employers or employees based on the proportion of immunization cost sharing (‘Vaccination Cost Sharing % by Employers’). No costs associated with vaccination were allocated to society.

b. All savings from *self-medication costs* were allocated solely to the employee.

c. Depending on how labor is compensated, the cost of sick time may be borne by the employer as reduced productivity, passed to the employee through reduced wages (as in the case of hourly-paid workers), or passed to an insurer (when absence is insured, for example with short-term disability). In this model, savings from avoided *absenteeism* was applied to the employer category.

d. All savings from *presenteeism* are credited to employers.

e. avings from avoided *outpatient and inpatient events* are split between employers and members, based on the benefit plan design.

f. The cost savings from avoided *death* were shared by all three stakeholders. Of the total VSL, approximately 75% was allocated towards societal savings, whereas the remaining 25% was apportioned to employers and employees. For employer groups particularly, a number of members will be dependents or could be retirees. Therefore, savings from avoided death was applied only to the members who were also employees. Therefore, this allocation is dynamic and based on enrollment input. The model assumes that 40% of members were employees, although this proportion could be adjusted. Employers were allotted savings equal to the product of (a) twice the annual salary (SAL) of an employee, (b) the proportion of the population who were employees, and (c) the age-weighted count of preventable deaths. This figure accounts for the loss productivity in an employee’s role until a new employee is hired and fully trained. For an employee, or rather, for the family of an employee, the loss of life accounts for projected loss of future earnings as well as the value to the family unit. This amount was allocated as estimated VSL multiplied by the percent of VSL that was allocated to the present value of future earnings (% PVFE) minus the employer’s portion. The remaining portion of the VSL reflected loss of the intrinsic value of life to society. The equations for each of these portions are:

*Employer* = age-weighted unit preventable death * 2 * SAL * % members employed

*Employee* = ($VSL * % PVFE) – Employer

*Society* = $VSL – Employer’s portion – Employee portion

Aggregate cost and savings estimates across all stakeholders, as well as an aggregate of employer (payer) and employees (members) savings are provided.

5. **Expected net savings** equaled total SAVINGS – total COSTS (i.e., 3–2).

6. **Expected net savings per vaccination (PV)** was the net savings total divided by the number of persons immunized (i.e., 5 / 1).

7. **Expected net saving per member per year (PMPY)** was the net savings total divided by the number of enrolled members.

## Results

**Figure 1:**
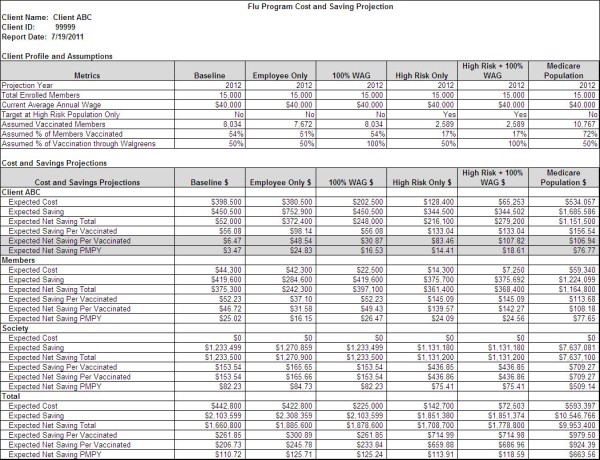
**Output from model comparing immunization strategies.** Note. WAG = immunization at Walgreens.

Five immunization scenarios were run for a fictitious company with 15,000 members and compared with a baseline (See Figure 1). As shown in the second column of Figure 1, the baseline scenario represented a population strategy (i.e., not high-risk) where 50% of individuals are vaccinated at pharmacies rather than a physician office. This strategy assumed vaccination of employees as well as their spouses and children. This baseline was compared to five possible immunization strategies:

1. Employee population only (i.e., not including dependents).

2. Full population vaccination with 100% at pharmacies

3. High risk individuals targeted with 50% at pharmacies.

4. High-risk individuals targeted with 100% vaccination at pharmacies.

5. Vaccination of seniors (65+) with 50% at pharmacies.

As described in the demographics assumption, the majority of population (60%) in the baseline scenario was assumed to be adults aged 18 to 64 years, with relatively few aged 65 and older (13%) and fewer than one in four under age 18 (26%). Assuming national age-specific coverage, 8,034 of 15,000 (54%) of individuals would be vaccinated.

Figure 1 presents the results of the baseline and other scenarios from the perspective of payers (Client ABC), members, society, and total (payer & member & society). The baseline model demonstrated that vaccinating all individuals in the sample population was cost effective from a payer perspective (expected net saving $6 PV; $3 PMPY) as well as from a member ($47 PV; $25 PMPY) and societal perspective ($154 PV; $82 PMPY). Total expect net savings to all stakeholder was estimated at $207 per vaccination.

Targeting employees (i.e., adults only) with at least 50% of employees being immunized at a pharmacy derived an employer cost savings of $49 per vaccination, compared to the baseline estimate of $6 per vaccination.

Increasing the proportion of vaccinations in pharmacies compared to PCP offices, increased the cost savings, as demonstrated by comparing the baseline and second scenarios in Figure 1. If the proportion of pharmacy-based immunizations is increased from 50% to 100%, the expected net savings for employers increased net savings per vaccination from $6 to $31.

Specifically targeting only high-risk individuals resulted in similar cost savings to pharmacy-based vaccination. Cost savings for employers was estimated to be $83 per vaccination for an at-risk strategy, compared to of $6 per vaccination for the population-strategy baseline. Cost savings were maximized by both targeting high-risk individuals and increasing the proportion of vaccination at pharmacies. When at-risk members were targeted and 100% of vaccinations occur in pharmacies, expected net savings increased to $108 per vaccination.

Individuals aged 65 and older represent a segment of persons deemed especially high risk by the CDC [16]. If framed from a health plan prospective, such as a Medicare Advantage (managed care) plan or retiree benefits plan, the expected net savings from immunizing 15,000 seniors yielded $107 per person vaccinated.

Confirming the findings from comparing scenarios, sensitivity analysis demonstrated that expected net savings for employers was most influenced by changes in age distribution of the target population (Range PMPY = [$-4.03, $106.94]), targeting at-risk populations ([$6.47, $83.46]), and the percent of vaccination in non-traditional settings [$-17.92, $30.87]). Results from the full sensitivity analysis are provided in Table2. Of note, positive net saving (PMPY = $2.83) was estimated even if the model assumed no risk of pandemic.

**Table 2:** Sensitivity analysis of employer expected net savings (ENS) per member per year (PMPY)

**Variable**	**Baseline Scenario**	**Input Range**	**Output Range**
Client Population Demographic	U.S. Average	[No children/seniors, Seniors only ]	[$-4.03, $106.94]
Risk level (Targeting High Risk)	No	[No, Yes]	[$6.47, $83.46]
Vaccination Channel:% in NTC	50%	[0%, 100%]	[$-17.92, $30.87]
Salary (Economic Variable)	$40 K	[$30 K, $60 K]	[$-1.69, $22.82]
Expected Cost of Immunization in TC	$82	[$60, $100]	[$16.48, $-1.52]
% Vaccine Cost Covered by Plan	90%	[80%, 100%]	[$11.99, $0.97]
Expected Cost of Immunization: NTC-TC extremes	$28:$82	[$23:$100, $41:$60]	[$0.72, $10.63]
% General Medical Cost Covered by Plan (Benefit Design)	78%	[60%, 90%]	[$1.07, $10.08]
Expected Cost of Immunization: NTC	$28	[$23, $41]	[$8.73, $0.62]
Risk of Pandemic	~2%	[0%, 4%]	[$2.83, $10.14]
Ratio Benefit to Wage (Benefit Design)	30%	[20%, 40%]	[$4.21, $8.75]
Projection Year	2012	[2011, 2013]	[$4.17, $8.92]
Average Percentage Vaccinated	~54%	[~44%, ~64%]	[$7.67, $5.69]

*Notes.* NTC = non-traditional channel; TC = traditional channel.

**Figure 2:**
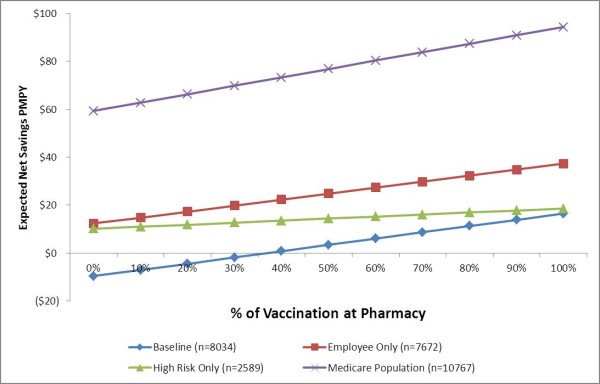
Opportunity analysis.

Figure 2 shows the change in employer savings that occurs when a larger share of the vaccinations are at pharmacies. When more than 37% of vaccinations were given at non-traditional, low cost settings, such as pharmacies, employers realized positive savings. All alternative strategies were more cost beneficial than baseline. The slope of cost benefit increases with the number of vaccinated persons, which causes the effect of decreasing vaccination to be more valuable in larger populations.

## Discussion

### Synopsis of key findings

This comprehensive model incorporated multiple sources of costs and benefits and allowed users to customize an immunization strategy according to the characteristics of the population. Depending on population, a broad immunization strategy was not always economically justified.

To evaluate the costs and benefits of offering vaccinations in alternative settings, vaccination in pharmacies were compared to the traditional vaccination setting of a primary care doctor’s office. Increasing the proportion of vaccination at pharmacies proportionally increased the cost savings of a modeled influenza immunization program. The primary mechanism for this savings was the elimination of the cost of an outpatient visit for the proportion of immunizations performed in a doctor’s office.

Compared to the baseline model, the ability to reach and vaccinate high-risk individuals increased cost savings. Likewise, immunizing seniors increased cost savings. A major driver of cost savings in these scenarios was avoiding serious complications, such as hospitalization and death, which are more prevalent in at-risk populations.

An important finding of this research was that universal immunization of an average U.S. population was cost beneficial from a payer perspective as long as 30% of vaccinations occur at low-cost, non-traditional settings such as pharmacies.

Furthermore, universal vaccination produced substantial cost savings from individual and societal perspectives.

### Comparison to relevant published work

The foundation of the model is a body of evidence-based assumptions from the published health literature. As expected, our results were consistent with findings from studies we used for our assumptions, such as Prosser et al. [12] and Molinari et al. [1]. For example, like Prosser et al., our model found that non-traditional setting increased cost savings for older and high-risk populations. However, a significant advantage of our model is the ability to actuarially adjust assumptions to fit requirements of different populations and to project to future years.

Based on the Behavioral Risk Factor Surveillance System 2010–11 survey, retail stores (including pharmacies) and workplaces provided 18.4% and 17.4% of influenza vaccinations, respectively [24]. On the other hand, doctors' offices provided 39.8% of vaccination. Assuming the results of this model are correct, opportunity exists for improving cost benefit of vaccination programs.

Although focused on workplace vaccinations, Lee et al. [13,25] developed a decision model for the value of for employers and found that, depending on the serologic attack rate, cost-savings varied from $15-$995 to $39-$1,494 per vaccinated employee. Lee et al. focused more on the importance of immunizing employees as a component of pandemic planning, and thus used higher estimates of influenza incidence (15% – 25%) compared with our default range (4.1% – 5.5%). Using the default parameters, our model is conservative from an employer cost perspective, providing a maximum savings estimate of $108 per vaccination for high-risk persons at pharmacies. Unlike our model, Lee et al. allocated all savings to employers rather than allocating any savings to society or employees. In fact, most of the published literature did not report costs and savings by various stakeholders. Bowen et al. [26] developed an online model of cost savings for preventive services, though not specific to influenza vaccination and only from the patient perspective.

Our results also echo those by Rothberg and Rose [27] who found that vaccination of working adults was cost saving under certain circumstances. Rather than comparing channels, Rothberg and Rose compared vaccination and anti-viral treatment to no treatment. In their model, lower vaccination cost or more than 2.4 days off work made vaccination more cost effective than the alternatives. Although our model did not consider anti-viral treatment, both models support immunization of working adults. Earlier research by Akazawa, Sindelar, and Paltiel [28] did not find vaccination of working-age individuals to be cost effective; however, as medical costs increase, avoiding illness and its related costs increasingly outweighs cost of preventive measures such as immunization. A 2001 model by Nichol [23] estimated a net savings of $13.66 (95% CI: -2.18, 32.97) per vaccinated person among health working adults. Similar to our results, a 2006 model by Maciosek and colleagues [29] concluded from their model that immunizing adults under age 65 was reasonably cost effective and immunizing adults age 65 and above was highly cost effective.

### Limitations of the model

Although the model incorporates a comprehensive set of variables that affect cost benefit of vaccination, the current model did not account for possible interactions between such variables [30]. Many assumptions were based on two Monte Carlo models in the published literature. Both of these models were based on the same retrospective cohort of medical claims in the mid-Atlantic U.S., and therefore, extrapolation to populations that vary significantly from this cohort would not be advised.

Although the use of value of the statistical life (VSL) is a standardized measure from the literature, VSL limited the ability of the model to adjust the costs of death to the average salary of the population. Absenteeism and presenteeism did not account for the possibility of exempt employees working extra hours later rather than losing compensation (i.e., using sick day).

Not all variables that influence cost benefit could be accounted for in the model. As discussed, our model did not compare immunization versus antiviral therapy. This model did not take into account the timing of vaccination within the season. For example, previous research [8] found that October was the most cost effective month to vaccinate older persons. In fact, delaying vaccination by even one month was estimated to increase both societal (by $400-490/1,000 persons) and third party payer costs (by $330-500/1,000 persons). However, they also found that vaccination up until February was still cost-effective. Therefore, vaccine interventions should strive to achieve the majority of vaccinations as soon as seasonal influenza vaccine is available and at least before February of a given influenza season.

### Implications for future research

Few articles were found that tested the accuracy of predictive outcomes of influenza vaccination cost models in actual practice. A recent Italian survey by Garattini and Koleva [31] demonstrated that vaccinated workers were less likely to be absent due to ILI, and thus validated this outcome of their model. While these findings from Italy are encouraging, validation of this model’s assumptions or outcomes in an U.S. employer group would strengthen support for its application.

## Conclusions

This paper described the development of an actuarial model to articulate the economic value of seasonal influenza vaccinations from various perspectives. Given the recent H1N1 pandemic, there is increasing awareness of the need for pandemic planning even in the private sector. The overriding objectives of the U.S. pandemic plan are “(1) limiting the burden of disease (i.e., morbidity and mortality); (2) minimizing social disruption caused by the pandemic; and (3) reducing economic losses attributable to the pandemic” [32]. Companies can support these goals by maximizing benefit of their immunization programs. This model can be used to help employers or health plans understand the impact of flu and vaccinations within their population. Furthermore, this decision model could provide policy makers with evidence-based value to compare immunization strategies and salient outcomes. Comparing various vaccination scenarios in this model confirms that targeting high-risk populations improves cost benefit of vaccination programs. Moreover, the model demonstrates that universal vaccination can be cost beneficial, especially when immunizations are provided in non-traditional settings such as pharmacies.

## Abbreviations

ILI, Influenza-like-illness; PV, Expected net saving per vaccinated; AHRQ, Agency for Healthcare Research and Quality; TC, Traditional channel; NTC, Non-traditional channel; ACIP, Advisory Committee on Immunization Practices PrP, Probability of a pandemic; ARA, Attack rate adjustment; OTC, Over-the-counter medication; VSL, Value of a statistical life; NPI, Number of persons immunized; PVFE, Present value of future earnings; PMPY, Expected net saving per member per year.

## Competing interests

This research was funded by Walgreens; the authors of this manuscript are employed or contracted by Walgreens.

## Authors’ contributions

IGD conceived of the study design and helped to draft the manuscript. MST conceived of the study design, interpreted data, and provided critical feedback for manuscript. JZ developed the model, analyzed the data, and critically revised the manuscript. HSK provided support for model development, interpreted data, and drafted the manuscript. All authors read and approved the final manuscript.
